# Development of next generation sequencing panel for *UMOD* and association with kidney disease

**DOI:** 10.1371/journal.pone.0178321

**Published:** 2017-06-13

**Authors:** Caitlin Bailie, Jill Kilner, Alexander P. Maxwell, Amy Jayne McKnight

**Affiliations:** Nephrology Research, Centre for Public Health, Queen’s University of Belfast, Belfast City Hospital, Belfast, Northern Ireland; The University of Tokyo, JAPAN

## Abstract

Chronic kidney disease (CKD) has a prevalence of approximately 10% in adult populations. CKD can progress to end-stage renal disease (ESRD) and this is usually fatal unless some form of renal replacement therapy (chronic dialysis or renal transplantation) is provided. There is an inherited predisposition to CKD with several genetic risk markers now identified. The *UMOD* gene has been associated with CKD of varying aetiologies. An AmpliSeq next generation sequencing panel was developed to facilitate comprehensive sequencing of the *UMOD* gene, covering exonic and regulatory regions. SNPs and CpG sites in the genomic region encompassing *UMOD* were evaluated for association with CKD in two studies; the UK Wellcome Trust Case-Control 3 Renal Transplant Dysfunction Study (n = 1088) and UK-ROI GENIE GWAS (n = 1726). A technological comparison of two Ion Torrent machines revealed 100% allele call concordance between S5 XL^™^ and PGM^™^ machines. One SNP (rs183962941), located in a non-coding region of *UMOD*, was nominally associated with ESRD (p = 0.008). No association was identified between *UMOD* variants and estimated glomerular filtration rate. Analysis of methylation data for over 480,000 CpG sites revealed differential methylation patterns within *UMOD*, the most significant of these was cg03140788 p = 3.7 x 10^−10^.

## Introduction

Chronic kidney disease (CKD) is defined by sustained and often progressive decrease in renal function (over months to years) which is generally irreversible. Renal function is most commonly measured by analysis of the serum creatinine concentration and then calculating an estimated glomerular filtration rate (eGFR) using formulae based on the individual’s age, gender, ethnicity and serum creatinine value [1]. Initially, CKD is typically asymptomatic and this can mean that diagnosis is delayed and CKD has progressed further before it is recognised [2]. With earlier diagnosis and effective management, CKD progression may be slowed or even halted [3]. CKD affects approximately 10% of adults however the prevalence in older age groups is much higher [4], and CKD is strongly associated with cardiovascular disease (cardiovascular events and hospitalisations) and premature mortality [5]. Individuals with CKD can progress to end-stage renal disease (ESRD), prompting the need for renal replacement therapies, such as chronic dialysis or renal transplantation. Treatments for CKD and ESRD represent a substantial socio-economic burden [6,7]. It is therefore important to identify earlier biological risk markers for CKD prediction as well as to highlight potential therapeutic targets.

Although there is evidence for a genetic predisposition to CKD, the risk alleles identified by genome-wide association studies (GWAS) are considered to contribute only a small proportion of overall complex disease risk [8,9,10,11,12]. CKD may be secondary to disorders such as diabetes, hypertension and glomerulonephritis [13,14,15,16]. Of interest, not all persons with diabetes, hypertension or glomerulonephritis will develop progressive CKD prompting investigation of genetic variants and epigenetic features which are associated with susceptibility to kidney disease or “protection” from developing this complication [17,18].

The *UMOD* gene encodes uromodulin protein (UMOD) that is synthesized solely in the epithelial cells of the thick ascending loop of Henle within the kidney. The role of UMOD in normal kidney function is incompletely understood, however, mutations of the *UMOD* gene are associated with autosomal dominant tubulointerstitial kidney disease (ADTKD-*UMOD*) which as previously been known as familial juvenile hyperuricaemic nephropathy (FJHN) and medullary cystic kidney disease (MCKD2), also known as UMOD-associated kidney diseases [19,20,21,22,23]. *UMOD* variants have also been identified as possible risk markers for more complex renal phenotypes such as CKD [24,25,26,27,28,29]. UMOD risk variants are associated with increased UMOD expression *in vitro* and *in vivo* [25]. For example, Olden and colleagues conducted meta-analysis of uromodulin levels in urine, highlighting SNPs in an LD block around the *UMOD* promoter, where the G allele of rs12917707 was consistently associated with decreased eGFR and up to a 2-fold increase in urinary excretion of uromodulin in a dose-dependent manner [28]. However, despite adequate power to identify a statistically significant association with variants in the *UMOD* gene, only a few genome-wide association studies for common renal phenotypes report *UMOD* as a top-ranked loci, suggesting associations may be restricted to particular population-specific phenotypes. Associations with ageing and age-related diseases such as diabetes and hypertension have also been suggested [30,31].

UMOD may have several “reno-protective” properties. The protein can form a gel-like permeability barrier in the nephron, possibly aiding with chemical concentration gradient maintenance [32]. The non-specific, carbohydrate structure of UMOD also allows it to bind and neutralize many molecules including urinary tract infection (UTI) causing bacteria and kidney stone forming calcium crystals [33,34]. Furthermore, UMOD may have a role in triggering an inflammatory response when the nephron has been damaged, thus recruiting immune cells [35] whilst, on the other hand, basal levels of UMOD may confer a protective effect in the event of acute ischaemic renal injury by decreasing inflammation [36]. Prolonged inflammation can trigger the development of progressive tissue fibrosis. This is in part mediated by up-regulation of transforming growth factor-beta (TGF-β) and recruitment of fibroblasts with resulting expansion of extracellular matrix (ECM) [37]. This progressive fibrotic process within the kidney is associated with pathological features such as glomerular sclerosis and tubulo-interstitial fibrosis which are correlated with clinical indicators of progressive CKD e.g. increased proteinuria and decreased eGFR.

*UMOD* is therefore an attractive biological candidate gene for both the development and progression of kidney disease. Previous studies on *UMOD* have examined selected exonic and upstream regions for variants that might be associated with CKD and eGFR. We have developed a next generation sequencing (NGS) panel that covers all coding and regulatory regions, within 2 kb upstream and downstream of *UMOD*. Published data was also reviewed and association evaluated between *UMOD* SNPs and ESRD. Additionally, DNA methylation data for the *UMOD* gene, were analysed for association with CKD.

## Methods

This study had full ethical approval from Queens University Belfast and the Office of Research Ethics Northern Ireland. PubMed was searched (search terms: UMOD, Uromodulin, Tamm-Horsfall protein, kidney disease, renal disease, Chronic Kidney Disease) to retrieve all publications relevant to *UMOD* SNPs and methylation data for any form of kidney disease (*last accessed [10 September 2015*]).

### Sequencing samples

**Fig 1:**
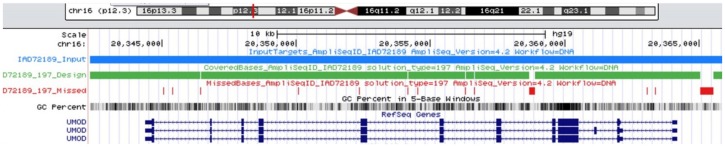
Ampliseq design. The UMOD gene region plus 2 kb upstream and downstream is highlighted by the blue line, coverage by Ampliseq amplicons is shown in green and regions not covered by the optimised assay design is shown in red. The structure of UMOD from RefSeq genes is represented in dark blue.

Existing high quality DNA was used for this study; DNA was originally extracted using the salting-out approach. For discovery of tag SNPs, 45 samples (23 transplant recipients with ESRD and 22 matched kidney donors with no known renal disease) were sequenced on an Ion Torrent Personal Genome Machine (PGM^™^) and 46 samples (23 transplant recipients with ESRD and 23 matched kidney donors with no known renal disease, including the 45 samples previously sequenced on the PGM) independently sequenced on the Ion Torrent 5S XL^™^ (Fig 1). This provided >95% power to detect all polymorphisms with minor allele frequency greater than 5%. All individuals were Caucasian and from the UK.

### AmpliSeq next generation sequencing panel design

Using http://www.ampliseq.com, a custom next generation sequencing panel was designed to sequence 23,928 bp, including 2 kb both upstream and downstream, of *UMOD* on chromosome 16p12.3. The design went through several iterations before achieving maximal coverage that was cost-effective.

### Ampliseq library preparation and NGS of *UMOD* gene

DNA libraries were prepared according to the Ampliseq Library Preparation Kit 2.0—96Lv (Thermo Fisher, San Diego, USA, MAN0006735) standard protocol using 20 ng of input DNA, with samples barcoded using Ion Xpress Barcodes 1–96 (Thermo Fisher). Prepared libraries were diluted to 26 pM using MilliQ water and combined into four samples pools, for sequencing on four 318 chips. Twenty-five microliters of each sample pool was enriched using a One-Touch Two (OT2) machine and automated enrichment system (Thermo Fisher) with the Ion PGM^™^ Template OT2 400 Kit (Thermo Fisher) to prepare Template-positive Ion Sphere Particles. Enriched products were prepared for sequencing using the Ion Personal Genome Machine^™^ (PGM) Hi-Q Sequencing Kit and run on the Personal Genome Machine using the Ion 318 chip v2 (Thermo Fisher, MAN0009816). In parallel, libraries for each sample were diluted to 70 pM and loaded onto an Ion Chef for sequencing on an Ion Torrent S5 XL^™^ using an Ion 530 chip (Thermo Fisher, MAN0010851) (Fig 1).

### Bioinformatic analysis for SNPs

Aligned BAM files from the PGM server was reformatted with SNP files generated and validated using PLINK [38], before using HaploView [39] software to assess the level of linkage disequilibrium to discern tag SNPs which could be used for optimised association analysis. Ion Torrent suite software version 4.6 aligned reads against the designed. BED file associated with the hg19 reference genome; data freely available from the authors on request. Using PARTEK genomics suite, data was mapped to hg19 and dbSNP138, filtered for log odds ratio ≥300 and total coverage at locus ≥20 to ensure reliable results, then annotated with rs# unique identifiers (if available) and functional effects. For the association analysis we used genome-wide genotyping information from two studies; the UK Wellcome Trust Case-Control 3 Renal Transplant Dysfunction Study (n = 1088; Affymetrix 6.0 array) comparing renal transplant recipients with ESRD to their matched donors with no kidney disease, and UK-ROI GENIE GWAS (n = 1726; Illumina OmniQuad array with data imputed to 1000 genomes) for controls with type 1 diabetes and no kidney disease compared to individuals with type 1 diabetes and kidney disease progressed to ESRD. PLINK [38] whole genome data analysis toolset was utilised to extract existing GWAS data for all SNPs between the first and last flanking SNPs from PARTEK analysis (rs9935655 to rs13333226 for Wellcome Trust data and rs4238595 and rs4293393 for GENIE data), within the GWAS datasets. We also conducted new association analysis using eGFR for non-ESRD samples in the UK-ROI GENIE collection. Characteristics of samples analysed in this study are presented in S1 Table. Nominal significance for association was set at p-value <0.05.

### 450K human methylation data analysis

Quantitative methylation data was previously generated using the Infinium Human Methylation 450K BeadChip (Illumina Inc.) according to manufacturer’s instructions for 255 cases with CKD and compared to 152 controls without kidney disease [17]. Stringent quality control was followed and adjustment for multiple testing applied to determine association between methylation patterns and CKD and methylation data extracted for *UMOD*, *UMODL1* and *UMODL1-AS1* genes.

## Results

### Next generation sequencing

Variants within the *UMOD* gene were determined for a total for 45 individuals; 23 kidney transplant recipients with ESRD, and 22 matching donors with no known kidney disease. Donor age ranged from 6–65 yrs and recipient age ranged from 11–65 yrs. Slightly more females were present in the recipient group compared to the donor group (seven and 12 respectively).

**Fig 2:**
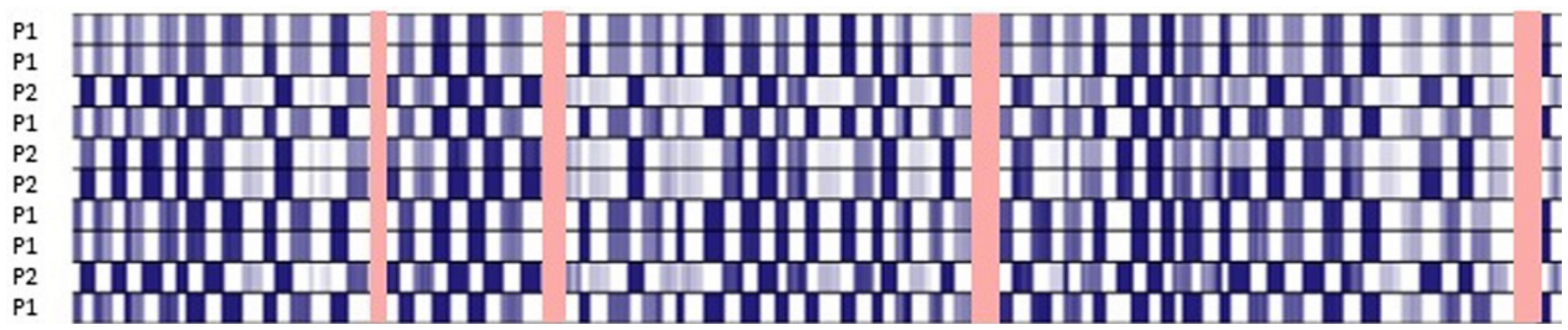
Coverage by amplicons. Sequence heat map from Partek Genomics Suite 6.6 showing complementary sequence coverage by pool 1 (P1) and pool 2 (P2) amplicons for pooled DNA. Areas not covered by either pool are highlighted in red.

The final AmpliSeq panel design employed 89 amplicons (125–375 bp) to cover 96.4% of the entire *UMOD* gene, starting at chromosome 16 position 20342322 and finishing at position 20366250 and including 100% coverage of exons. This panel covers all mutations currently screened by the UK Genetic Testing Network (*ukgtn*.*nhs*.*uk*) or published (Fig 1). An average of 445,650 mapped reads per sample was observed using the PGM^™^ (range 237,240–912,642 reads) of which >97% were on target, providing a mean depth of 2,052. Two primer pools were used to ensure that overlapping amplicons would optimally multiplex (45 amplicons in pool 1 and 44 amplicons in pool 2) (Fig 2).

During quality control, results from two amplicons were removed as they did not meet the minimum required reads (AMPL7158733025 failed with an average of 42 reads per sample; AMPL7158732941 performed poorly with an average of 92 reads per sample) from the PGM data; following removal, an average of 2,498 reads per amplicon was observed.

**Fig 3:**
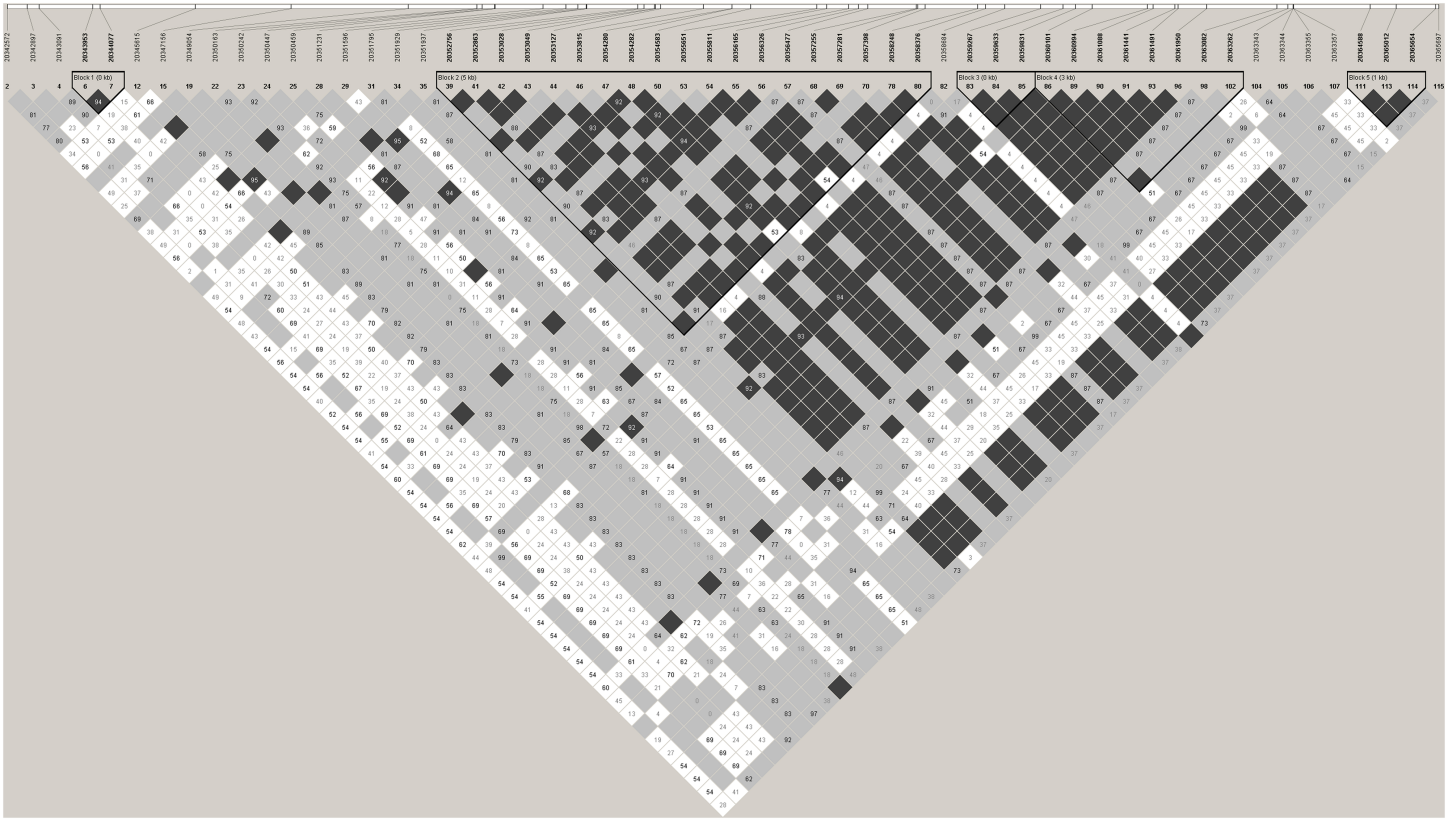
Linkage disequilibrium plot using D' confidence intervals. Linkage disequilibrium plot for all SNPs with minor allele frequency >5% and in Hardy-Weinberg Equilibrium. Colour scheme for D' confidence interval plot: Strong evidence of LD (dark grey), Strong evidence of recombination (white), Uninformative (light grey).

**Fig 4:**
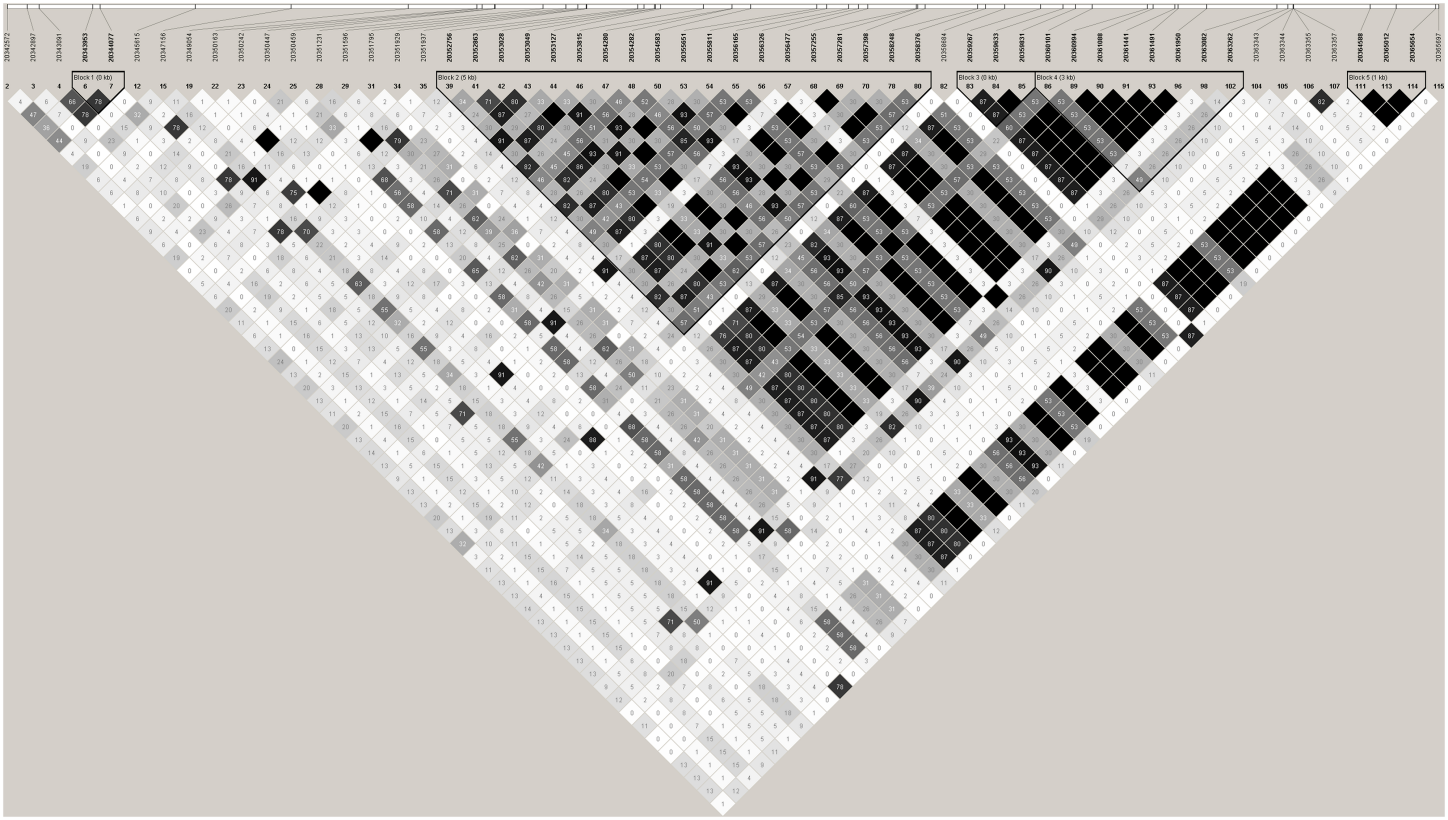
Linkage disequilibrium plot using r^2^. Linkage disequilibrium plot for all SNPs with minor allele frequency >5% and in Hardy-Weinberg Equilibrium. Colour scheme for r^2^ plot as follows: r^2^ = 0 (white), 0 < r^2^ < 1 (shades of grey), r^2^ = 1 (black).

**Fig 5:**
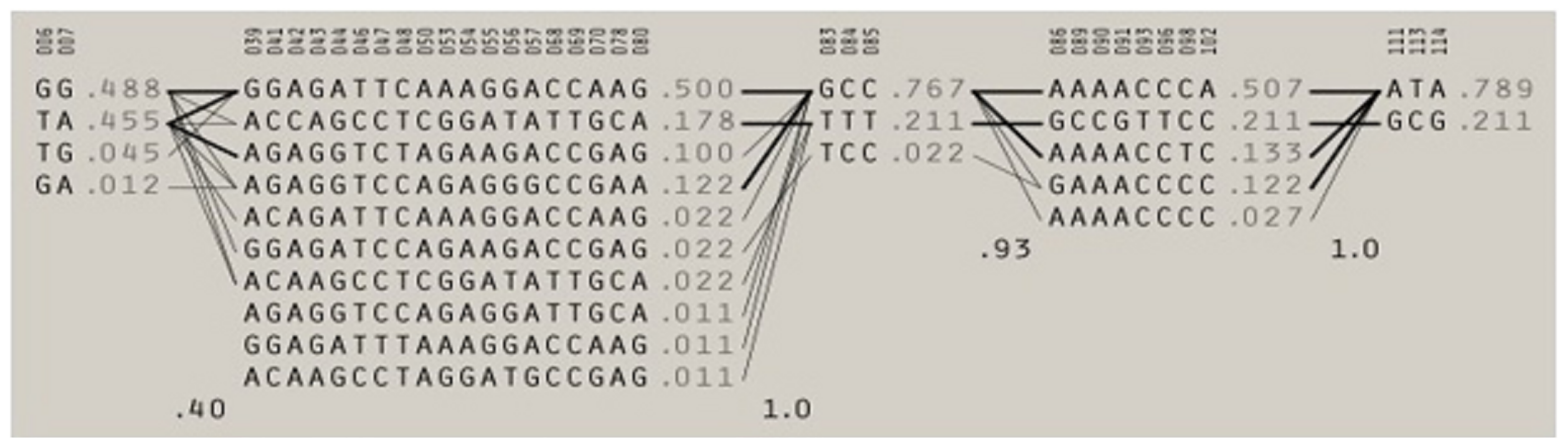
Haplotype blocks. Haplotype blocks with population frequencies displayed next to each haplotype. Lines represent the most common crossings between block (thicker lines are more common) beneath which the value of multiallelic D’ is shown, measuring the level of linkage disequilibrium (LD) between blocks. Greater recombination is denoted by a value closer to zero.

In total we found 119 genetic variants within our population of 23 cases with ESRD and 22 controls without renal disease; 29 of these were insertions, deletions or multiallelic polymorphisms (S2 Table). Genotype completion obtained for most variants was 100%; one variant (20363262) showed 91% genotype completion. Of the remaining 90 SNPs, 60 were observed with minor allele frequency greater than 5% (S1 Fig). Using the tagger option in HaploView revealed that 20 SNPs, in 21 tests, captured 100% of the alleles at r^2^˃0.8 (mean r^2^ = 0.97) and are thus sufficient for association analysis in larger cohorts (Figs 3, 4 and 5).

**Fig 6:**
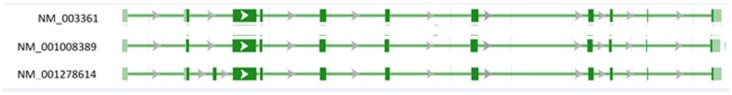
UMOD transcripts. Three UMOD transcripts (3'-5') from NCBI gene website [last accessed: 17/10/2015]. *Green blocks indicate the position of exons on the sequence with arrows denoting the direction of expression*.

Initial PARTEK analysis revealed a total of 3,317 differences from the reference sequence across all samples (n = 45) where log odds ratio ≥5. 116 unique variants were discovered in the *UMOD* region with log odds ratio ≥ 300 and at least 20 reads of which 78 had rs identifiers in dbSNP build 138. The functional effect of SNPs differs depending on which *UMOD* transcript is considered; for example, one variant at 20352618 (rs55772253) in our discovery cohort exerted a non-synonymous effect on different exons depending on the transcript (Table 1; Fig 6).

**Table 1:** Chromosome location and alleles associated with multiple transcripts and associated gene locations for non-synonymous SNP rs55772253.

Chromosome	Position	Reference allele	Alternate allele	Transcript	Gene Section	Functional Effect	Nucleotide Change	Amino Acid change
*Chr 16*	20352618	C	A	NM_001008389 NM_003361	Exon 7 Exon 7	Missense	c.1372G>T	V458L
*Chr 16*	20352618	C	A	NM_001278614	Exon 8	Missense	c.1471G>T	V491L

**Fig 7:**
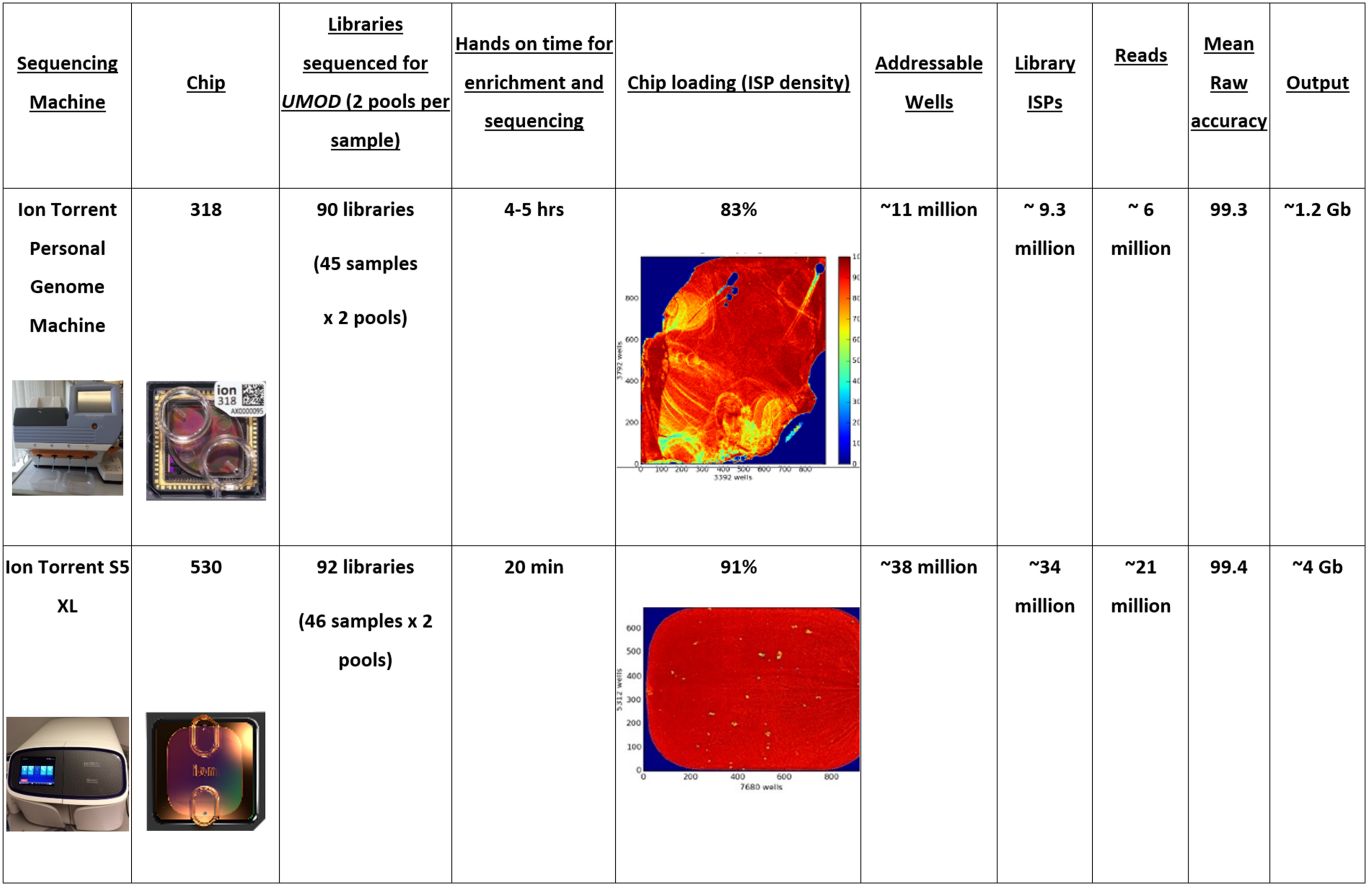
Comparison of suitability of PGM and S5XL next generation sequencers for analysis of UMOD.

An average of 195,205 mapped reads per sample was observed using the S5 XL^™^ (range 100,081–258,626 reads) of which >98% were on target, providing a mean depth of 965. Comparative results obtained from 318 and 530 chips (Fig 7) confirm both PGM and S5 XL are suitable for this analysis, with the choice of machine optimally guided by the number of samples to be sequenced.

### Association analysis

Ninety-two SNPs were extracted from the UK/ROI GEnetics of Nephropathy—an International Effort GWAS study (GENIE) and seven SNPs were extracted from the Wellcome Trust Case-Control 3 Renal Transplant Dysfunction Study (WTCCC3-RTD) study (two of these SNPs were not found in GENIE) (S3 Table). Only one SNP extracted from the GENIE study, was nominally significantly associated with ESRD (rs183962941: p = 0.008). This SNP was not in linkage disequilibrium (LD) with any previously reported associated variants. Four other variants, extracted from the GENIE study, showed a trend towards significance, (rs191101580, rs187087030, rs8060932, rs8062123), and rs8060932 is in LD with another SNP (rs4238595) associated with FJHN [21]. Our study did not reveal any association with eGFR for non-ESRD samples with or without diabetes. No SNPs in the WTCCC3-RTD study revealed any significant association with ESRD or eGFR.

### Methylation data analysis

Methylation status was determined for 485,577 CpG sites in a total of 407 individuals. Significant cg sites were extracted from the large dataset for *UMOD*, *UMOL1* and *UMODL1-AS1*. Following stringent quality control and analysis differential methylation of three cg sites was observed in *UMOD* revealing cg03140788 as the most significant with a p-value of 3.7x10^-10^ (Table 2). Two related genes that are also expressed in the kidney; *UMODL1* and *UMODL1-AS1*, were explored, revealing eight associated CpG sites, with genome wide significance; P_max_ = 2.9x10^-32^ (cg16624482) was the most significant for non-diabetic ESRD. A list of SNPs that could influence probe binding and thus affect DNA methylation is provided in S4 Table.

**Table 2:** Showing sites within UMOD, UMODL1 and UMODL1-AS1 that are significantly associated with CKD.

Symbol	Description	CpG site	Features	Adj. P-value
*UMOD*	Uromodulin	cg03140788	Body; CpG Island	3.7 x 10^−10^
		cg07817806	3’UTR	4.4 x 10^−6^
		cg06861044	Body; CpG Island	6.6 x 10^−5^
*UMODL1*	Uromodulin-Like 1	cg16624482	Body; S_Shore	2.90 x 10^−32^
		cg21935742	TSS200	8.10 x 10^−31^
		cg09727148	3'UTR	2.10 x 10^−28^
		cg23931796	TSS200	4.70 x 10^−27^
		cg00785029	Body	3.20 x 10^−8^
		cg03643948	Body; Island	8.90 x 10^−12^
		cg01542693	Body; S_Shore	2.40 x 10^−9^
		cg03240473	Body; Island	1.50 x 10^−12^
*UMODL1-AS1*	Uromodulin-Like 1- Anti-Sense 1	cg01542693	TSS200	2.40 x 10^−9^
		cg03240473	Body; Island	1.50 x 10^−12^

## Discussion

**Fig 8:**
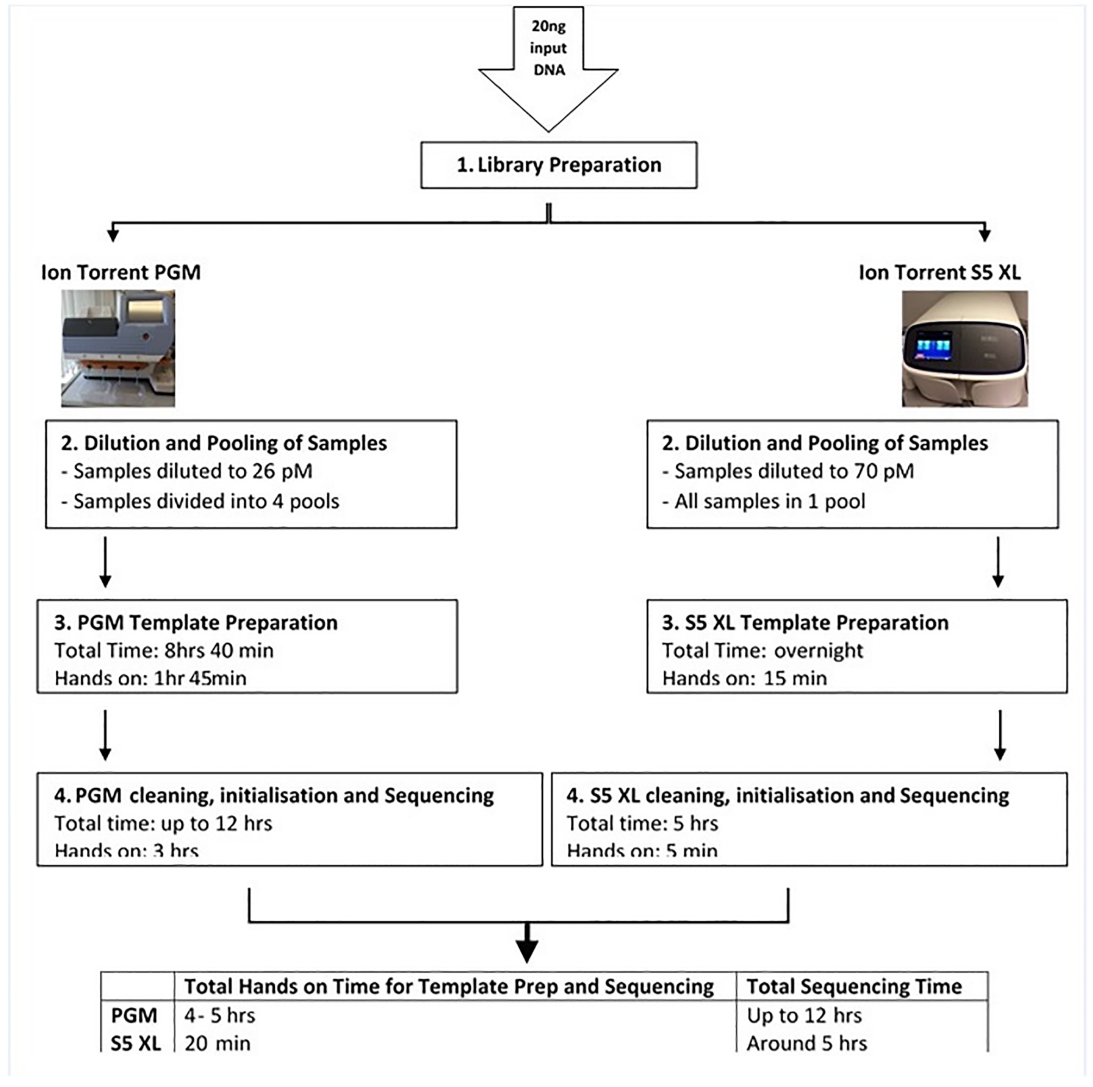
Sequencing workflow. Workflow for Ion Torrent PGM and S5 XL showing hands on time and total time for template preparation and sequencing.

This study aimed to develop a next generation sequencing (NGS) assay for the detection of variants in the *UMOD* gene, covering both coding and non-coding regions. A cost-effective approach for targeted sequencing or comprehensive genotyping of individuals and larger population-based cohorts was developed using ≤20 ng of input DNA (Fig 8).

We observed one SNP, rs183962941 nominally associated with ESRD (p = 0.008), which has not been previously reported and was not shown to be in LD with any other variant using the SNP Annotation and Proxy Search (SNAP—Broad Institute [*last accessed*: *14 September 2015*] [40]). Located in the intronic region between exons 6 and 7 of *UMOD*, the potential function of rs183962941 remains to be determined; searching the Blood eQTL browser [41], the GTEx Portal [42], or HaploReg v4 [43] does not reveal functional associations with this SNP, however it should be noted this SNP is not present on common Illumina or Affymetrix genotyping arrays, HapMap CEU release 22, or 1000 genomes pilot 1 according to SNAP [40]. Of the 116 SNPs annotated in PARTEK analysis, 91 were identified within intronic and / or potential regulatory regions, which may be important to help create a better risk profile for kidney disease.

Four other variants were extracted from the GENIE study, which were trending towards significance at the five percent level (rs191101580, rs187087030, rs8060932, rs8062123); one of these (rs8060932; p = 0.08) was shown to be in LD with another SNP, rs4238595 (r^2^ = 0.89), associated with FJHN [21]. rs13333226 has been previously reported to be associated with reduced urinary uromodulin excretion as well as lower risk of hypertension and cardiovascular disease [13], however there was no evidence of association in this cohort. rs13333226 is also in LD with the most common SNP for *UMOD* (rs12917707), which has been reported to be associated with CKD, eGFR and ESRD [8,9,11,26,28].

Two non-synonymous SNPs were identified from a variant at position 20352618 (rs55772253), resulting in an amino acid change V458L or V491L depending on the *UMOD* transcript. V458L was previously reported by Kottgen *et al*. [24] who suggested it was associated with decreased eGFR and concentration of uromodulin; this SNP is ‘probably damaging’ (score 0.998) according to prediction software SIFT [44] and PolyPhen-2 [45].

The significant differential methylation pattern in *UMOD* (cg03140788 p = 3.7 x 10^−10^) was detected in a gene body CpG island (CGI). Jones *et al*. [46] suggest that methylation of a gene body CGI may be a method of controlling transcription of a gene that has two promoters (alternative promoter usage) by stopping transcription from this site while still allowing transcription and elongation from the earlier promoter.

Association was also identified in *UMODL1* and *UMODL1-AS1*. *UMODL1* was first characterised in 2004, but there has been little work published on this gene, with only 11 articles in PubMed returned from a search of ‘*UMODL1*’, and one returned from a search of ‘*UMODL1 and kidney*’ (February 2017). This gene is expressed in the kidney [47] and genome-wide association analysis of 3,851 individuals revealed association with *UMODL1* and CKD [48]. *UMODL1-AS1* is a long non-coding RNA, which has been little studied to date, but has potential to provide prognostic information for CKD.

We observed 100% SNP concordance between the S5 XL^™^ and PGM^™^ in SNPs with a quality score greater than 100. A visual review of highlighted differences between TorrentSuite-based SNP calling from the two sequencers revealed a microsatellite and homopolymer region (eight A/T) which generated experimental artefacts. Both PGM^™^ and S5 XL^™^ are ideally suited for targeted SNP and indel detection, while S5 XL^™^ represents a significant cost saving in terms of labour and consumables compared to using PGM^™^.

This study combines genetic and epigenetic investigations to improve understanding of the genetic architecture of the *UMOD* gene region. Strengths of the study included its significant power to identify 95% of variants with MAF >5% in 45 carefully phenotyped individuals, as well as the replication on the S5 XL^™^ sequencer and 100% concordance of SNPs between both next generation sequencers. However, tens of thousands of individuals will be required to evaluate rare variants in this gene to provide adequate power to identify significant associations for rare SNPs. A potential limitation to this study was that all samples were Caucasian and so variants that might be present within other ethnicities were not assessed.

In summary, there is sufficient evidence to suggest variants at the *UMOD* locus are associated with Mendelian and more common complex CKD aetiologies; larger, more comprehensive studies are warranted. Many SNPs that have been significantly associated with CKD are only nominally associated with ESRD if at all, perhaps suggesting different roles of action or the need for more carefully phenotyped cohorts. Large observational CKD studies such as GEnetics of Nephropathy: an International Effort (GENIE) [49], Chronic Renal Insufficiency Cohort (CRIC) [50] and German Chronic Kidney Disease Study (GCKD) [51] will allow more insight into the development of ESRD, and hopefully lead to the identification of a more comprehensive risk profile for both CKD and ESRD.

## Supporting information

S1 Table Characteristics of samples analysed in this study. (PDF)

S2 Table SNPs uniquely identified from sequencing data. (PDF)

S3 Table List of SNPs uniquely extracted either in the UK/ROI GEnetics of Nephrology an International Effort cohort or Wellcome Trust Case- Control 3 Renal Transplant Dysfunction Study cohort. (PDF)

S4 Table SNPs that could influence probe binding for certain cg sites in UMOD and UMODL1. Highlighted in bold font are cg sites that had significant p-values.
(PDF)

S1 Fig SNPs depicted as blue lines across the gene structure with exons noted. (PDF)
