# Supplementary material for: Development of next generation sequencing panel for UMOD and association with kidney disease
Source: PLoS One. 2017 Jun 13;12(6):e0178321. doi: 10.1371/journal.pone.0178321 (PMC5469457; doi:10.1371/journal.pone.0178321)
Supplement: S1 Table — (PDF) [file pone.0178321.s001.pdf]

Development of next generation sequencing panel for *UMOD* and association with kidney disease

Caitlin Bailie[1], Jill Kilner[1], Alexander P Maxwell[1], Amy Jayne McKnight[1*]

1. Nephrology Research, Centre for Public Health, Queen's University of Belfast, Belfast, BT9 7AB, Northern Ireland,
AJM*a.j.mcknight@qub.ac.uk

S1 Table: Characteristics of samples analysed in this study

	case	*control*
Age (mean (SD), y)	*41.6 (17.2)*	*37.8 (17.1)*
Sex (% male)	*62*	*56*
Ethnicity (% White)	*100*	*100*
ESRD (%)	*60*	*0*
Primary renal diagnosis (%)	*100*	*0*
Diabetes (with proteinuria)	*72 %*	*0*
Glomerulonephritis	*6 %*	*0*
Chronic pyelonephritis / tubulointerstitial disease	*6 %*	*0*
ADPDK	*5 %*	*0*
CKD not separately classified	*11%*	*0*
