# Supplementary material for: Development of next generation sequencing panel for UMOD and association with kidney disease
Source: PLoS One. 2017 Jun 13;12(6):e0178321. doi: 10.1371/journal.pone.0178321 (PMC5469457; doi:10.1371/journal.pone.0178321)
Supplement: S2 Table — (PDF) [file pone.0178321.s002.pdf]

Development of next generation sequencing panel for *UMOD* and association with kidney disease

Caitlin Bailie[1], Jill Kilner[1], Alexander P Maxwell[1], Amy Jayne McKnight[1*]

1. Nephrology Research, Centre for Public Health, Queen's University of Belfast, Belfast, BT9 7AB, Northern Ireland,
AJM*a.j.mcknight@qub.ac.uk

S2 Table: SNPs identified from next generation sequencing data.

RSID	Position
rs73541287	20342425
None	20364382
None	20351836
None	20356810
None	20356105
None	20363052
None	20351397
None	20356814
rs12444268	20342572
None	20358205
rs9935655	20342897
rs4238595	20343091
None	20349779
rs74457213	20343627
rs8062123	20343953
rs8060932	20344077
rs111699931	20344532
rs199562069	20344735
rs1123670	20345615
rs4635355	20347156
None	20343703
None	20343706
None	20343793
None	20343876
rs72776658	20348995
rs72776659	20349054
rs117849751	20349109
rs8044650	20350163
rs6497474	20350242
rs111624876	20350447
rs71384446	20350459
rs146641336	20350468
None	20350084
None	20350085
rs8051744	20351134
rs11859916	20351231

None	20350259
rs9923532	20351596
rs200680486	20351796
rs55906116	20351815
rs72776660	20351929
rs7198000	20351937
rs141800038	20352532
rs55772253	20352618
rs9646256	20352756
rs141733080	20352840
rs9928757	20352863
rs9928936	20353049
rs4780884	20353127
rs75459600	20353370
rs60136849	20353815
rs7498751	20354280
rs6497475	20354282
rs78913673	20354691
rs8054296	20355651
None	20353503
None	20353504
None	20353690
rs34262842	20355811
rs11647727	20356165
rs34356953	20356326
None	20354601
rs73541299	20356477
None	20355238
rs189908977	20356806
None	20355918
None	20356010
None	20356012
rs184978356	20356828
rs12934320	20357255
rs12934455	20357281
None	20356655
None	20356726
None	20356730
rs4506906	20357398
rs367785364	20357415
rs375846119	20357601
rs114112267	20358082
rs112146268	20358213
rs368035737	20358221
rs9928003	20358248
None	20358209

rs7203451	20358376
rs114333799	20358404
rs7204210	20358684
rs28640218	20359267
rs28544423	20359633
rs13335818	20359831
rs7193058	20360101
rs116892778	20360907
rs142717731	20360919
None	20360683
None	20360729
rs34882080	20361441
rs11865380	20361464
rs35650857	20361491
rs36060036	20361950
rs75645968	20362106
None	20361606
rs7189301	20363082
rs143657120	20363197
None	20363051
