# Supplementary material for: Development of next generation sequencing panel for UMOD and association with kidney disease
Source: PLoS One. 2017 Jun 13;12(6):e0178321. doi: 10.1371/journal.pone.0178321 (PMC5469457; doi:10.1371/journal.pone.0178321)
Supplement: S3 Table — (PDF) [file pone.0178321.s003.pdf]

Development of next generation sequencing panel for *UMOD* and association with kidney disease

Caitlin Bailie[1], Jill Kilner[1], Alexander P Maxwell[1], Amy Jayne McKnight[1*]

1. Nephrology Research, Centre for Public Health, Queen's University of Belfast, Belfast, BT9 7AB, Northern Ireland,
AJM*a.j.mcknight@qub.ac.uk

S3 Table: List of SNPs uniquely extracted either in the UK/ROI GEnetics of Nephrology an International Effort cohort or Wellcome Trust Case- Control 3 Renal Transplant Dysfunction Study cohort.

Study	Rs ID	Position	P-value
**UK ROI GENIE**	rs183962941	20354447	0.0076
	rs191101580	20345714	0.05
	rs187087030	20345455	0.08
	rs8060932	20344077	0.08
	rs8062123	20343953	0.08
	rs4238595	20250592	0.08
	rs141800038	20352532	0.1
	rs193056167	20356816	0.1
	rs142717731	20360919	0.1
	chr16:20344278:I	20344278	0.1
	rs4238595	20343091	0.1
	rs143321062	20361646	0.2
	rs77875418	20360359	0.2
	rs59027126	20361326	0.2
	rs79245268	20362115	0.2
	chr16:20348692	20348692	0.2
	rs187459570	20347206	0.2
	rs140845467	20363614	0.3
	rs7204210	20358684	0.3
	rs6497474	20350242	0.3
	rs182342188	20358827	0.3
	rs76563024	20363839	0.4
	rs184944834	20343366	0.4
	rs72776660	20351929	0.4
	rs116892778	20360907	0.4
	rs6497475	20354282	0.5
	rs7195581	20356824	0.5
	rs1123670	20345615	0.5
	rs74936567	20360777	0.5
	rs113852396	20344568	0.5
	rs75459600	20353370	0.6
	rs183747797	20356943	0.6
	rs7193058	20360101	0.6
	rs114112267	20358082	0.6
	rs114333799	20358404	0.6

rs116040334	20350612	0.6
rs75645968	20362106	0.6
rs117738531	20362285	0.6
chr16:20363045:I	20363045	0.7
rs143657120	20363197	0.7
rs11647727	20356165	0.7
chr16:20361087:I	20361087	0.7
rs74457213	20343627	0.7
rs7189301	20363082	0.7
chr16:20356721:D	20356721	0.7
rs112433356	20361858	0.7
rs73541299	20356477	0.7
rs9646256	20260257	0.7
rs11647727	20263666	0.7
rs28544423	20267134	0.7
rs13335818	20267332	0.7
rs7198000	20351937	0.8
rs111699931	20344532	0.8
rs71384446	20350459	0.8
rs9922248	20363262	0.8
chr16:20358347:I	20358347	0.8
rs111624876	20350447	0.8
rs7203451	20358376	0.8
rs35650857	20361491	0.8
rs13335818	20359831	0.8
chr16:20360993:I	20360993	0.8
rs34882080	20361441	0.8
rs7498751	20354280	0.8
rs11859916	20258732	0.8
SNP16-20260119	20260119	0.8
rs4506906	20357398	0.9
rs8054296	20355651	0.9
rs36060036	20361950	0.9
rs55906116	20351815	0.9
rs4293393	20364588	0.9
rs4780884	20353127	0.9
chr16:20353027:I	20353027	0.9
rs28544423	20359633	0.9
rs9928757	20352863	0.9
rs111658337	20347346	0.9
rs11859916	20351231	0.9
rs9928003	20358248	0.9
chr16:20344735	20344735	0.9
rs8044650	20350163	0.9
rs12934320	20357255	0.9
rs9935075	20346545	0.9

	rs72776658	20348995	0.9
	rs34356953	20356326	0.9
	rs55772253	20352618	0.95
	rs72776659	20349054	0.95
	rs9923532	20351596	0.95
	rs4635355	20347156	0.96
	rs9928936	20353049	0.96
	chr16:20351814:D	20351814	0.97
	rs189908977	20356806	0.98
	rs34262842	20355811	0.98
	rs184978356	20356828	0.98
	rs186684841	20354179	0.98
	rs60136849	20353815	0.99
	rs28640218	20359267	0.99
	rs12934455	20357281	0.99
	rs9646256	20352756	1.0
	rs111484604	20346180	1.0
	rs1123670	20253116	1.0
**WTCCC - RTD**	rs9935655	20250398	0.2
	rs13333226	20273155	0.2
