# Supplementary material for: Development of next generation sequencing panel for UMOD and association with kidney disease
Source: PLoS One. 2017 Jun 13;12(6):e0178321. doi: 10.1371/journal.pone.0178321 (PMC5469457; doi:10.1371/journal.pone.0178321)
Supplement: S4 Table — Highlighted in bold font are cg sites that had significant p-values. (PDF) [file pone.0178321.s004.pdf]

Development of next generation sequencing panel for *UMOD* and association with kidney disease

Caitlin Bailie[1], Jill Kilner[1], Alexander P Maxwell[1], Amy Jayne McKnight[1*]

1. Nephrology Research, Centre for Public Health, Queen's University of Belfast, Belfast, BT9 7AB, Northern Ireland,
AJM*a.j.mcknight@qub.ac.uk

S4 Table: SNPs that could influence probe binding for certain cg sites in UMOD and UMODL1. Highlighted in bold font are cg sites that had significant p-values.

Cg site	Gene	SNP	Distance	Minor Allele Frequency
cg00382053	UMOD	rs112166439	40	0.5
cg06294373	UMOD	rs112787476	21	0.5
**cg06861044**	UMOD	rs78691203	34	0.07
**cg07817806**	UMOD	rs113468667;rs111699931	19;45	0.5;0.5
cg17812431	UMOD	rs114632195;rs75124070	16;1	0.06;0.02
cg21996068	UMOD	rs112787476	10	0.5
cg00349542	UMODL1	rs111996953	16	0.5
cg00969162	UMODL1	rs115842277	4	0.05
**cg01542693**	UMODL1	rs73905547;rs60147771;rs57966423	1;26;28	0.5;0.25;0.33
cg02489157	UMODL1	rs220118;rs34632081;rs113572114	0;23;43	0.38;0.40;0.50
cg03441713	UMODL1	rs79477845	37	0.04
cg03643948	UMODL1	rs117212334;rs116188869	11;2	0.04;0.03
cg07638368	UMODL1	rs11203185	1	0.47
cg08880261	UMODL1	rs220159	1	0.001
**cg16624482**	UMODL1	rs73373640;rs116673538	42;20	0.50;0.02
cg21004633	UMODL1	rs220272;rs220273	20;35	0.19;0.17
cg22706458	UMODL1	rs58524852;rs113242448;rs67503452; rs74443976;rs56743498	0;15;38;38;42	0.17;0.5;0.5; 0.5;0.25
cg23782413	UMODL1	rs113320317	33	0.5
**cg23931796**	UMODL1	rs79846567	1	0.04
cg24977306	UMODL1	rs73905568	2	0.5
cg25587200	UMODL1	rs79477845	27	0.04
