# Supplementary material for: Development of next generation sequencing panel for UMOD and association with kidney disease
Source: PLoS One. 2017 Jun 13;12(6):e0178321. doi: 10.1371/journal.pone.0178321 (PMC5469457; doi:10.1371/journal.pone.0178321)

Development of next generation sequencing panel for *UMOD* and association with kidney disease

Caitlin Bailie[1], Jill Kilner[1], Alexander P Maxwell[1], Amy Jayne McKnight[1*]

1. Nephrology Research, Centre for Public Health, Queen's University of Belfast, Belfast, BT9 7AB, Northern Ireland,
AJM*a.j.mcknight@qub.ac.uk

S1 Fig: SNPs depicted as blue lines in the context of exons in UMOD on the chromosome.

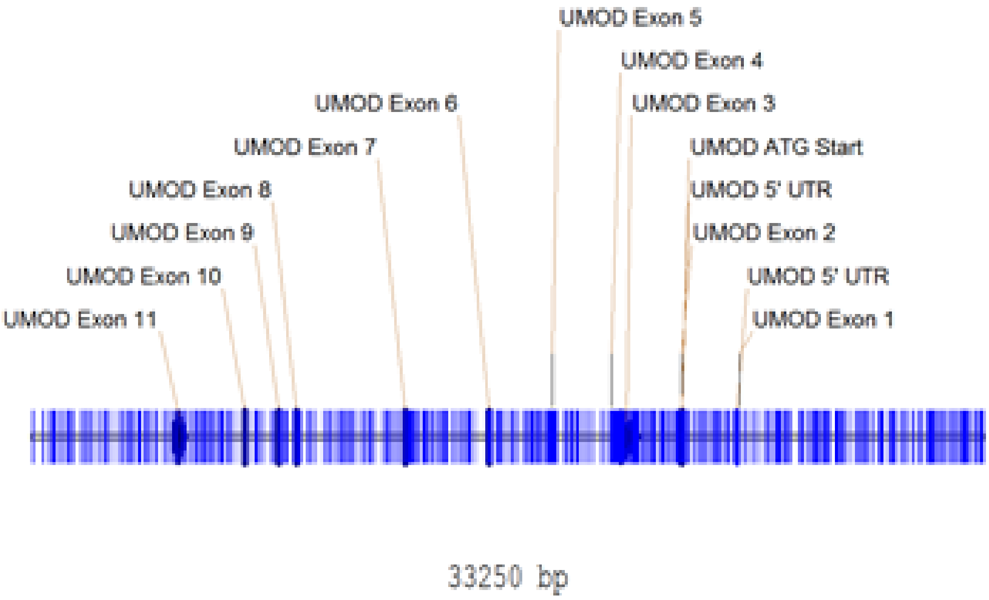

Supplement: S1 Fig — (PDF) [file pone.0178321.s005.pdf]
